# Antibacterial effects and resistance induction of silver and gold nanoparticles against *Staphylococcus aureus*‐induced mastitis and the potential toxicity in rats

**DOI:** 10.1002/mbo3.698

**Published:** 2018-08-05

**Authors:** Ayman Elbehiry, Musaad Al‐Dubaib, Eman Marzouk, Ihab Moussa

**Affiliations:** ^1^ Department of Bacteriology Mycology and Immunology Faculty of Veterinary Medicine University of Sadat City Sadat City Egypt; ^2^ Department of Public Health College of Public Health and Health Informatics Qassim University Buraidah Saudi Arabia; ^3^ Department of Veterinary Medicine College of Agriculture and Veterinary Medicine Qassim University Buraidah Saudi Arabia; ^4^ Department of Medical laboratories College of Applied Medical Science Qassim University Buraidah Saudi Arabia; ^5^ Department of Botany and Microbiology College of Science King Saud University Riyadh Saudi Arabia

**Keywords:** antibacterial, nanoparticles, resistance induction, *Staphylococcus aureus*, toxicity

## Abstract

*Staphylococcus aureus* (*S. aureus*) is one of the prevalent mastitis‐inducing pathogens worldwide. The resistance of *S. aureus* to antibiotics is a common issue for dairy farms. Recently, nanoparticles (NPs) have been used for treating antibiotic‐resistant bacteria. We therefore aimed to investigate the antimicrobial effect of silver and gold NPs (AgNPs and AuNPs, respectively) and the resistance developed by *S. aureus* as well as the toxic effects of both NPs in rats. We used 198 *S. aureus* strains to determine the antibacterial effects of AgNPs and AuNPs. The microdilution method was used to establish the minimum inhibitory concentrations (MICs) of both NPs. To induce resistance, 20 *S. aureus* strains were passaged 10 times in broth medium with sublethal doses of NPs and an additional 10 times without NPs to examine the stability of resistance. Histopathology was performed after oral administration to the rats with the study doses of 0.25, 0.5, 1, and 2 mg/kg of NPs for 30 days. The MICs of 10‐nm AgNPs, 20‐nm AgNPs, 10‐nm AuNPs, and 20‐nm AuNPs against *S. aureus* were 14.70 ± 1.19 μg/ml, 9.15 ± 0.13 μg/ml, 24.06 ± 2.36 μg/ml, and 18.52 ± 1.26 μg/ml, respectively. Most strains developed strong resistance when treated with 20‐nm or 10‐nm AgNPs, whereas only two strains were resistant to 10‐nm AuNPs and three strains to 20‐nm AuNPs. No cross‐resistance between NPs and various antibiotics was identified in any of the adapted *S. aureus* strains. Organ histopathology revealed that 0.25, 0.5, and 1 mg/kg doses of AgNPs and AuNPs were not toxic to rat tissue. In contrast, a higher dose (2 mg/kg) of NPs impaired all organs tested. This study demonstrates the antibacterial effects of NPs. *S. aureus* strains develop resistance less frequently against AuNPs than AgNPs, and neither AuNPs nor AgNPs was toxic to rats at low doses.

## 1 INTRODUCTION

Bovine mastitis is one of the major problems facing the dairy industry worldwide (Benić, Habrun, & Kompes, 2012; Elbehiry, Al‐Dubaib, Marzouk, Osman, & Edrees, 2016; Preethirani et al., 2015). Several bacterial species are responsible for bovine mastitis infections, *Staphylococcus aureus* (*S. aureus*) being one of the most prevalent mastitis pathogens, causing the most virulent forms of bovine mastitis (El Behiry, Schlenker, Szabo, & Roesler, 2012; Yang et al., 2008). Furthermore, milk infected with *S. aureus* raises a public health alarm from consumers because *S. aureus* produces various types of enzymes and toxins that can lead to serious food‐borne illnesses (Johler et al., 2013). *S. aureus‐*induced mastitis causes a number of problems including a sharp decrease in milk production, reproductive complications in the cow, and expenses incurred from replacing corrupted milk and increased labor (Jamaran & Zarifm, 2016).

The resistance of mastitis‐causing *S. aureus* to antibacterial drugs is well‐known to dairy farmers (Pitkälä, Haveri, Pyörälä, Myllys, & Honkanen‐Buzalski, 2004). Consequently, current research is focused on developing new types of safe, effective, and affordable antibacterial agents to combat *S. aureus* (Dehkordi, Fatemeh, & Azizollah, 2011; Jamaran & Zarifm, 2016). Nanotechnology has enabled the use of nanoparticles (NPs) for the treatment of antimicrobial‐resistant bacteria (Koo, Rubinstein, & Onyuksel, 2005; Kumar, Curtis, & Hoskins, 2018; Lopez Goerne, Alvarez Lemus, Morales, López, & Ocampo, 2012). The strength antimicrobial NPs’ properties results from their large surface area‐to‐volume ratio, which also reduces the likelihood of antibiotic tolerance. NPs ranging from 10 to 100 nanometers (nm) in size are thought to possess unique physical and chemical features (Boschi & De Sanctis, 2017; Sadeghi, Jamali, Kia, Amininia, & Ghafari, 2010; Zhang, Gao, Zhang, & Bao, 2001).

Recently, NPs have been used as an alternative method for treatment of various antibiotic‐resistant bacterial infections and may solve the problem of multidrug‐resistant bacteria (Franci et al., 2015; Rai, Deshmukh, Ingle, & Gade, 2012; Wang, Hu, & Shao, 2017); in particular, silver nanoparticles (AgNPs) have received a great deal of attention (Salomoni, Léo, & Rodrigues, 2015; Szmacinski et al., 2008). Silver has been previously utilized as an antimicrobial agent against multiple types of bacteria (Lazar, 2011; Taraszkiewicz, Fila, Grinholc, & Nakonieczna, 2013) because of its low cytotoxicity (Biel et al., 2011). Recently, AgNPs have exhibited antimicrobial activity against *S. aureus* and are also highly effective against methicillin‐resistant *S. aureus* (MRSA) (Wady et al., 2014). The advantages of using AgNPs as antimicrobial agents include their extensive range of activity, cost effectiveness, and high efficacy (Patra & Baek, 2017).

The bactericidal properties of AgNPs have been studied previously (Taraszkiewicz et al., 2013). Various reports indicate that AgNPs’ total surface area is larger than that of larger silver particles, and therefore AgNPs are more effective as antimicrobial agents than silver particles. A recent discovery showed that the antimicrobial action of gold nanoparticles (AuNPs) is also enhanced by their larger total surface area per unit volume (Sreekanth, Nagajyothi, & Lee, 2012; Zhang, Shareena‐Dasari, Deng, & Yu, 2015); therefore, AuNPs have better antibacterial action than gold alone (Fayaz et al., 2011; Mihai & Malaisteanu, 2013). Various researchers have reported the antibacterial action of AuNPs against human microorganisms, but the minimum inhibitory concentration (MIC) results for *Escherichia coli* and *S. aureus* were not statistically significant for 7‐nm and 16‐nm AuNPs (Ali, Thajuddin, Jeganathan, & Gunasekaran, 2011; Shamaila et al., 2016; Zhou, Kong, Kundu, Cirillo, & Liang, 2012). Recent studies have noted that many types of microorganisms cannot develop resistance to silver and AgNPs treatment, which are considered antimicrobial agents for multidrug‐resistant bacteria (Mahmoudi & Serpooshan, 2012; Rai et al., 2012), although other studies dispute this claim (Gupta, Maynes, & Silver, 1998).

As NPs can kill bacteria by direct adhesion with their cellular walls without the need to pierce the cells, most mechanisms of antibiotic resistance are irrelevant. This increases the expectation that NPs are less likely to resist bacteria than antibiotics (Wang et al., 2017). In general, the antimicrobial mechanism of action of NPs is explained as adhering to one of three models: oxidative stress induction (Gurunathan, Han, Dayem, Eppakayala, & Kim, 2012), metal ion release (Nagy et al., 2011), or nonoxidative mechanisms (Leung et al., 2014). These three types of mechanisms can occur at the same time. However, continued exposure to sublethal doses of biocides can lead to the overexpression of efflux pumps and bacterial resistance may develop (Gilbert & McBain, 2003).

Few studies have been conducted on microorganisms toward evaluating whether their resistance to sublethal doses of NPs also results in antibiotic resistance (El Behiry et al., 2012). However, while some studies propose that a cross‐resistance between NPs and antibiotics can develop, others indicate no such link exists (McDonnell & Russell, 1999; Russell, Tattawasart, Maillard, & Furr, 1998). Because NPs are extensively dispersed in air, medicine (nanodevices and nanostructures), and even food, the toxicological effect of NPs is a major issue in nanotechnology (Kong, Seog, Graham, & Lee, 2011). Although the potential adverse effects of NPs have increasingly attracted attention, no reliable trials have been conducted (Kong et al., 2011; Stone, Johnston, & Schins, 2009). Only a few researchers have analyzed the toxicological effects of NPs on the gastrointestinal tract, and they found evidence of toxicity (Sardari et al., 2012).

In addition, biomedical researchers have demonstrated that several medical devices release silver ions into the bloodstream, and these ions accumulate in different organs (e.g., liver, spleen, and kidney), which can cause severe toxicity and ultimately death (Park et al., 2010; Sardari et al., 2012). It is assumed that AgNPs and AuNPs can lead to toxicity, but the mechanism of their cytotoxicity remains unclear (Tang et al., 2009). However, Wen et al. (2017) demonstrated that accumulation of AgNPs in the spleen, liver, lungs and kidneys of rats proposing the AgNP transmitted and accrued into specific target organs where they may further generate Ag^+^. Moreover, the significant increase in chromosomal damage and polyploidy cell rates implied the possible genotoxicity of AgNP. In trials with various metal NPs, AgNPs have exhibited greater toxicity than other metals, including iron, nickel, manganese, and aluminum (Braydich‐Stolle, Hussain, Schlager, & Hofmann, 2005).

Given the previously mentioned data, we believe that studying the antimicrobial and harmful effects of NPs is a vital and urgent matter. Our study aims to evaluate the in vitro of and resistance to AgNPs and AuNPs in *S. aureus*; and to assess the in vivo toxicity of both NPs after 30 days of oral administration in rats.

## 2 MATERIALS AND METHODS

### 2.1 Bacterial strains

This study used 198 *S. aureus* strains recovered from 418 quarter milk samples collected from five dairy herds with a high prevalence of *S. aureus* in Al‐Qassim region, Saudi Arabia. Isolation was conducted based on the recommendations of the National Mastitis Council for the investigation of milk samples.

### 2.2 Identification of *S*. *aureus* strains

The tube coagulase test and the Vitek^™^ 2 compact system (BioMe′rieux, Paris, France) were used for the phenotypic identification of all *S. aureus* strains. Simplex polymerase chain reaction (PCR) was used for genotypic identification. The amplifications were performed with three primer sets: one specific for an *S. aureus*‐specific region of the nuc gene, which encodes a thermonuclease; another for mecA, a determinant of methicillin resistance; and the last for a genus‐specific 16S rRNA sequence that was used as an internal amplification control for staphylococcal DNA. Proteomic identification with the Microflex LT system (Bruker Daltonics, Bremen, Germany) was used as a confirmatory method of identification.

### 2.3 Nanoparticles used in the study

Two aqueous colloidal NP solutions were purchased from PlasmaChem GmbH (Berlin, Germany) for use in our study (Table 1). Chemical reduction method was used for synthesis of both NPs. In brief, AgNPs were synthesized by using silver nitrate as a source of silver, sodium borohydride solution as a reducing agent, and polyvinylpyrrolidone (PVP) as a stabilizing agent to prevent particle agglomeration (Wang, Qiao, Chen, Wang, & Ding, 2005). The silver nitrate was dissolved in deionized water to keep the impurity levels of the NPs low, then sodium citrate tribasic hydrate and PVP were added. A sodium borohydride solution was added to the mixture and stirred for 30 min. The formation of AgNPs was observed by changing the color solution from colorless to brown. Synthesize of AuNPs was also carried out by a reduction of 10‐mM tetrachloroauric acid (HAuCl_4_) using sodium citrate (Sigma‐Aldrich, USA). Briefly, an aqueous solution of HAuCl_4_.3H_2_O was boiled under reflux while being stirred. Changing the color of the solution from yellow to deep red after adding 10‐ml trisodium citrate (1%) indicated the formation of spherical AuNPs. The solution was refluxed for 20 min, then left to cool at 25°C. Afterward, the solution was filtered through a 0.45‐μm acetate filter and stored at 4°C. Morphology of the synthesized NPs was examined by transmission electron microscopy (Oberkochen, Germany). NPs size distribution was measured according to the dynamic light scattering using a Malvern zeta sizer Nano ZS^®^ device (Sysmex, Nederland).

**Table 1:** Concentration of silver and gold nanosized particles

Nanoparticle	Particle size, nm	Concentration
Silver	10	0.1 mg/ml
	20	0.05 mg/ml
Gold	10	0.05 mg/ml
	20	0.05 mg/ml

### 2.4 Antibacterial activity of AgNPs and AuNPs against *S. aureus*

The antibacterial effect of AgNPs and AuNPs against 198 *S. aureus* strains was evaluated at the MICs of the NPs, which were achieved by the broth microdilution method. In brief, twofold serial dilutions (200–0.39 μg/ml) were prepared. Each well was consequently filled with 100 μl of inoculum and the MICs were recorded after 24 hr of incubation at 37°C. To evaluate the antibacterial action of AgNPs and AuNPs on bacterial growth, the MICs for all isolates were determined after 24 hr using the optical density (OD_600_) of the bacterial culture solutions. The final cell concentrations of bacterial inoculants were 10^6^–10^7^ CFU/ml. The concentration of AgNP and AuNP that inhibited the growth of ≥50% of bacteria was recognized as the LD_50_, compared with the growth in the treatment‐free well.

To determine the minimum bacterial concentration (MBC), 50 μl aliquot of wells that did not illustrate turbidity was transferred in Tryptose Soya Agar (TSA) plates which are not supplemented with NP and then incubated at 37°C for 24 hr. All plates were examined before and after incubation for presence or absence of bacterial growth. Plates which displayed no growth of bacteria indicate that the concentration of NP was lethal. The number of surviving organisms was determined by viability counts. The lowest concentration of NP that inhibited the growth of ≥99.99%% of *S. aureus* was defined as MBC. All of the experiments were duplicated on two different days. *S. aureus* American Type Culture Collection (ATCC) 25923 and *S. aureus* ATCC 29213 were utilized as positive controls.

### 2.5 Induction of *S. aureus* resistance to AgNPs and AuNPs

Twenty *S. aureus* strains that had been previously exposed to AgNPs and AuNPs were used in this experiment. The strains with low MIC values were chosen to induce resistance. Resistance induction for *S. aureus* strains was attempted through frequent passaging of these strains in growth media with sublethal doses of NPs below the MIC, at concentrations where the strains still showed growth. Therefore, the concentrations used in the current trial for 10‐nm AgNPs, 20‐nm AgNPs, 10‐nm AuNPs, and 20‐nm AuNPs were 3.12 μg/ml, 1.56 μg/ml, 6.25 μg/ml, and 3.12 μg/ml, respectively. The MICs for each NP were compared before and after 10 passages. Under completely sterile conditions, the isolates were passaged 10 times in TSB with sublethal doses of both AgNPs and AuNPs at 3‐day intervals. The MIC values after the 10th passage of all isolates were recorded and compared with the MIC values before passage. The culture purity was tested by streaking the bacteria into medium specific for *S. aureus* (Baird‐Parker Agar). The stability of NP resistance was evaluated by continuing subcultures of the resistant isolates every day for 10 passages in NP‐free nutrient broth. The final MIC was determined after the 10th passage. Throughout this experiment, culture purity testing was conducted.

### 2.6 Assessment of cross‐resistance between NPs and antibiotics

Based on the M7‐A8 guidelines of the Clinical Laboratory Standards Institute (2009), the broth microdilution method was used to evaluate cross‐resistance to Senstitre Antibiotic Plates (TREK Diagnostic Systems, UK). Briefly, *S. aureus* strains were streaked onto Mueller‐Hinton Agar plates (Sigma Aldrich, USA) and then incubated for 18–24 hr at 37°C. Around four to five *S. aureus* colonies were transferred to sterile tubes containing 5 ml of 0.9% NaCl solution.

By using the Senstitre Nephelometer (TREK Diagnostic Systems, UK), the turbidity of the growing broth culture was adjusted (ca 1 × 10^5^ KbE/ml). Subsequently, 11 ml of Mueller‐Hinton broth was inoculated with 15 μl of the modified TSB and 50 μl of the mixture was inoculated into each well of the Senstitre plates. The plates were covered with foil and incubated at 37°C for 24 hr. The Senstitre Automatic Reader (TREK Diagnostic Systems, UK) was used to read the plates.

### 2.7 Toxicological effect of AgNPs and AuNPs in rats

To test the adverse effects of AgNPs and AuNPs in vivo, 200 male adult albino rats (8–12 weeks old with 180 ± 20 g body weight) were used in this study. The rats were kept in steel wire cages (5/cage) in the veterinary research laboratory, College of Agriculture and Veterinary Medicine, Qassim University. The rats were divided into five groups (40/group): G1, G2, G3, G4, and G5. G5 was the control group and received 0.9% NaCl solution throughout the experiment. The other four groups, G1 to G4, were experimental groups that were further divided into four subgroups (10/group) according to the study dose of NPs.

Under strict hygienic conditions, the rats were fed a normal diet. All groups had free access to water and were kept at 22 ± 3°C. Two weeks before the experiment, the rats were acclimated to the research laboratory conditions. The rats were administered with AgNPs (10 and 20 nm) or AuNPs (10 and 20 nm) for 4 weeks (once/day, 7 days/week). The following equation was followed for the oral administration of 0.25, 0.5, 1, and 2 mg/kg (study doses) of nanosized particles:

$$\text{Administered dose}\;=\;\frac{\text{Body weight}\;\;\times \;\;\text{study dose}}{\text{Conc. of nano‐sized particles}\;\;\times \;\;1000}$$

After 4 weeks, the rats were euthanized by cervical dislocation under ketamine‐xylazine anesthesia. The tissue organs were detached, washed with 0.9% NaCl, and inspected for morphology. Subsequently, the organs were stabilized in 10% buffered formalin and reserved for histopathological examination. A tissue processing device (Sakura Tissue Tek VIP E300) was used to process all tissue samples before paraffin block preparation. After paraffin embedding, 5 μm sections were cut and stained with hematoxylin and eosin (H and E) for histopathological investigation. All procedures in this study were carried out according to the National Institute of Health Guide for the Care and Use of Laboratory Animals and the Ethical Rules of the Experimental Animal Care Centre, Qassim University, KSA.

### 2.8 Statistical methods

The data obtained from antibacterial susceptibility testing were imported into the Statistical Package for the Social Sciences (SPSS), and all estimations were carried out using SPSS version 20.0 (SPSS Inc., Chicago, IL, United States).

## 3 RESULTS

### 3.1 Identification of *S. aureus*

All 198 *S. aureus* strains gave positive results in the tube coagulase test, and 184 *S. aureus* strains were properly identified by the Vitek^™^ 2 compact system as 123 methicillin‐sensitive *S. aureus* (MSSA) strains and 61 MRSA strains. Genotypic identification was performed using simplex PCR and the results revealed that the 16S rRNA and nuc genes were found in all of the *S. aureus* strains, while the mecA gene was found in the 66 *S*. MRSA strains. Microflex LT results showed that 132 of the MSSA isolates and 66 of the MRSA isolates were correctly identified, with score values ranging from 2,000 to 3,000. Based on a visual analysis of mass regions, several variations were observed that allowed us to discriminate between MSSA and MRSA. The strongest area of variable signals (3,800–5,900 Da) exhibited distinguishable intensities between the MSSA and MRSA strains: higher peak intensities at 3,993 Da, 4,121 Da, and 5,845 Da were observed in MRSA but not in MSSA.

### 3.2 The antibacterial effect of AgNPs and AuNPs against *S. aureus* mastitis

Using the serial dilution method, the mean MIC, MIC_50_, and MIC_90_ values of AgNPs and AuNPs against *S. aureus* isolates were determined and are summarized in Table 2. The mean MIC values of 10‐nm AgNPs, 20‐nm AgNPs, 10‐nm AuNPs, and 20‐nm AuNPs were 14.70 ± 1.19 μg/ml, 9.15 ± 0.13 μg/ml, 24.06 ± 2.36 μg/ml, and 18.52 ± 1.26 μg/ml, respectively. The MIC range of the AgNPs (10–20 nm) varied from 3.12 to 25 μg/ml, whereas this range was 6.25–50 μg/ml for the AuNPs (10–20 nm). The MIC_50_ values of the 10‐nm AgNPs, 20‐nm AgNPs, 10‐nm AuNPs, and 20‐nm AuNPs were 6.25 μg/ml, 3.12 μg/ml, 12.5 μg/ml, and 6.25 μg/ml, respectively, whereas the MIC_90_ values for these agents were 12.5 μg/ml, 6.25 μg/ml, 25 μg/ml, and 25 μg/ml, respectively. These findings indicate that all isolates were susceptible to the tested NPs, and low concentrations of NPs were needed to kill the *S. aureus* isolates.

**Table 2:** MICs (μg/ml) for AgNPs and AuNPs against *S. aureus*

Nanoparticle	Mean MIC	MIC range	MIC50	MIC90
Silver, 10 nm	14.70 ± 1.19	6.25–25	6.25	12.5
Silver, 20 nm	9.15 ± 0.13	3.12–25	3.12	6.25
Gold, 10 nm	24.06 ± 2.36	12.5–50	12.5	25
Gold, 20 nm	18.52 ± 1.26	6.25–50	6.25	25

**Figure 1:**
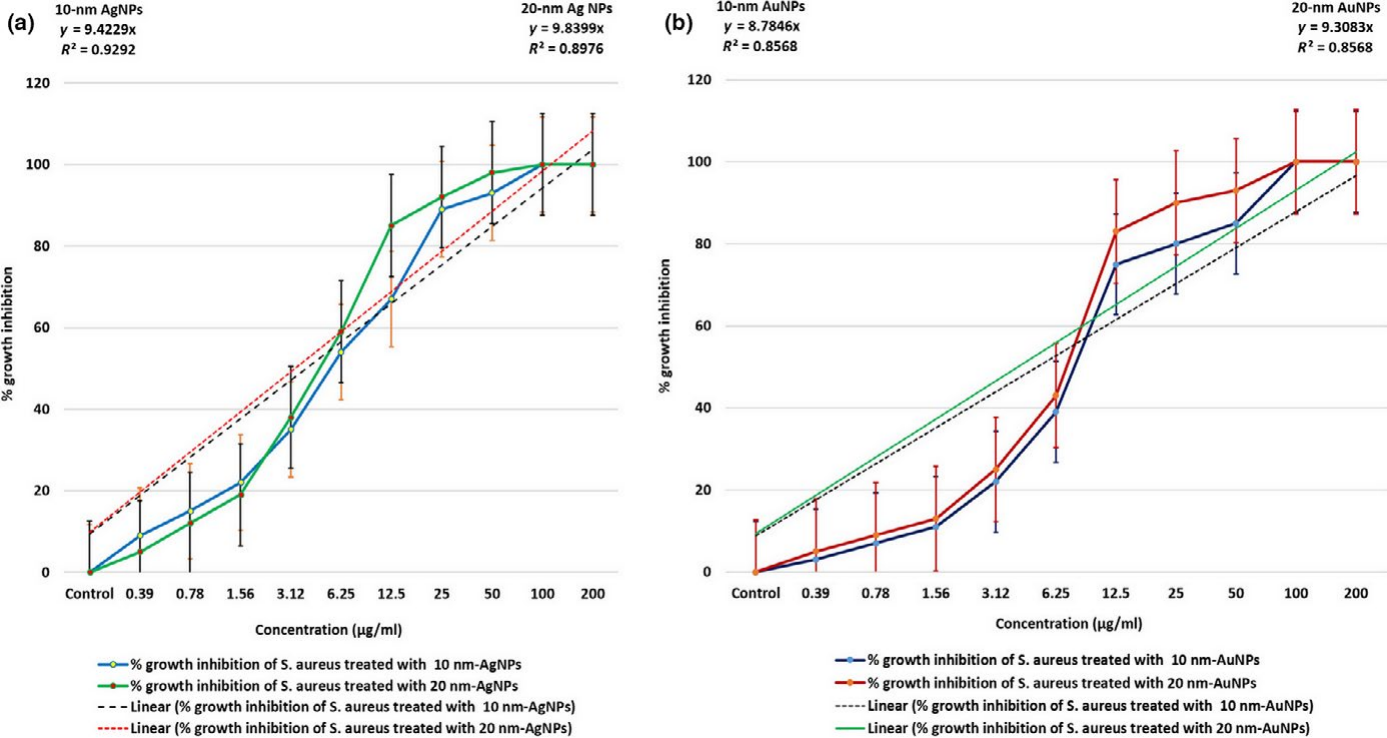
Antibacterial activity of AgNPs and AuNPs against *Staphylococcus aureus* (data represent mean ± *SE* of double replicates). (a) Graphical representation of % growth inhibition over concentration of 10‐ and 20‐nm AgNPs and (b) % growth inhibition over concentration of 10‐ and 20‐nm AuNPs

The in vitro antibacterial susceptibility of AgNPs (10–20 nm) and AuNPs (10–20 nm) was measured in terms of growth rates of *S. aureus* using turbidimetric growth analysis over a concentration range of NPs from 0.39 to 200 μg/ml (Figure 1a and b) with percentage growth inhibition curve against concentration range along with a linear regression coefficient between the two plotted parameters. We statistically analyzed the MBC for both NPs and found that the growth for all *S. aureus* strains was completely inhibited at concentrations of 100 μg/ml and 200 μg/ml when compared to 0.39–50 μg/ml. A significant correlation between concentration range and percent of growth inhibition with *R*‐squared value (*R* ^2^) values equal to 0.929, 0.897, and 0.8568 for *S. aureus* treated with 10‐nm AgNPs, 20‐nm AgNPs, and 10‐ to 20‐nm AuNPs, respectively.

### 3.3 Resistance developed by *S. aureus* against AgNPs and AuNPs

Twenty *S. aureus* strains were tested against low concentrations of AgNPs and AuNPs to determine whether resistance to NPs can be induced in these strains over time. The MIC values detected for the 20 *S. aureus* strains subjected to successive sublethal doses of NPs are shown in Table 3. Four *S. aureus* strains (S3, S7, S14, and S20) demonstrated strong resistance to the 10‐nm AgNPs, and resistance to 20‐mm AgNPs was detected in 10 strains. In contrast, only two strains (S5 and S15) developed resistance to the 10‐nm AuNPs, and only three strains (S5, S15, and S20) showed strong resistance to the 20‐nm AuNPs.

**Table 3:** MICs (μg/ml) for AgNPs and AuNPs with sublethal doses against *S. aureus*

Strain	AgNPs								AuNPs							
	Conc. below MIC		MIC before passage		MIC after 10th passage		MIC after 10th stable passage		Conc. below MIC		MIC before passage		MIC after 10th passage		MIC after 10th stable passage	
	10 nm	20 nm	10 nm	20 nm	10 nm	20 nm	10 nm	20 nm	10 nm	20 nm	10 nm	20 nm	10 nm	20 nm	10 nm	20 nm
S1	3.12	1.56	6.25	3.12	6.25	6.25	6.25	6.25	6.25	3.12	25	6.25	6.25	6.25	25	6.25
S2	3.12	1.56	12.5	12.5	6.25	6.25	12. 5	12.5	6.25	3.12	25	25	25	25	25	25
S3	3.12	1.56	12.5	3.12	25	12.5	25	12.5	6.25	3.12	12.5	25	12.5	12.5	12.5	12.5
S4	3.12	1.56	6.25	3.12	6.25	6.25	6.25	6.25	6.25	3.12	12.5	6.25	12.5	6.25	12.5	6.25
S5	3.12	1.56	6.25	6.25	3.12	6.25	6.25	6.25	6.25	3.12	12.5	6.25	50	50	50	50
S6	3.12	1.56	12.5	6.25	12.5	6.25	12.5	6.25	6.25	3.12	25	25	25	25	12.5	25
S7	3.12	1.56	6.25	3.12	12.5	6.25	12.5	6.25	6.25	3.12	25	12.5	12.5	6.25	12.5	6.25
S8	3.12	1.56	6.25	6.25	6.25	6.25	6.25	6.25	6.25	3.12	12.5	25	12.5	25	12.5	25
S9	3.12	1.56	12.5	3.12	6.25	3.12	6.25	3.12	6.25	3.12	50	6.25	50	6.25	50	6.25
S10	3.12	1.56	6.25	3.12	6.25	6.25	6.25	6.25	6.25	3.12	50	50	50	50	50	50
S11	3.12	1.56	12.5	12.5	6.25	3.12	6.25	3.12	6.25	3.12	25	25	25	12.5	25	25
S12	3.12	1.56	6.25	3.12	6.25	6.25	6.25	6.25	6.25	3.12	12.5	12.5	12.5	12.5	12.5	12.5
S13	3.12	1.56	6.25	3.12	6.25	6.25	6.25	12.5	6.25	3.12	12.5	12.5	12.5	12.5	12.5	12.5
S14	3.12	1.56	12.5	6.25	50	25	50	25	6.25	3.12	25	25	25	12.5	12.5	12.5
S15	3.12	1.56	6.25	12.5	6.25	12.5	6.25	12.5	6.25	3.12	25	12.5	50	50	50	50
S16	3.12	1.56	12.5	6.25	12.5	12.5	6.25	12.5	6.25	3.12	25	25	25	25	25	25
S17	3.12	1.56	6.25	12.5	6.25	12.5	6.25	6.25	6.25	3.12	25	25	25	12.5	25	12.5
S18	3.12	1.56	6.25	3.12	6.25	6.25	12.5	6.25	6.25	3.12	12.5	12.5	12.5	6.25	12.5	12.5
S19	3.12	1.56	6.25	6.25	6.25	6.25	6.25	6.25	6.25	3.12	25	12.5	25	12.5	25	12.5
S20	3.12	1.56	12.5	12.5	25	25	25	25	6.25	3.12	50	12.5	50	25	50	25

**Figure 2:**
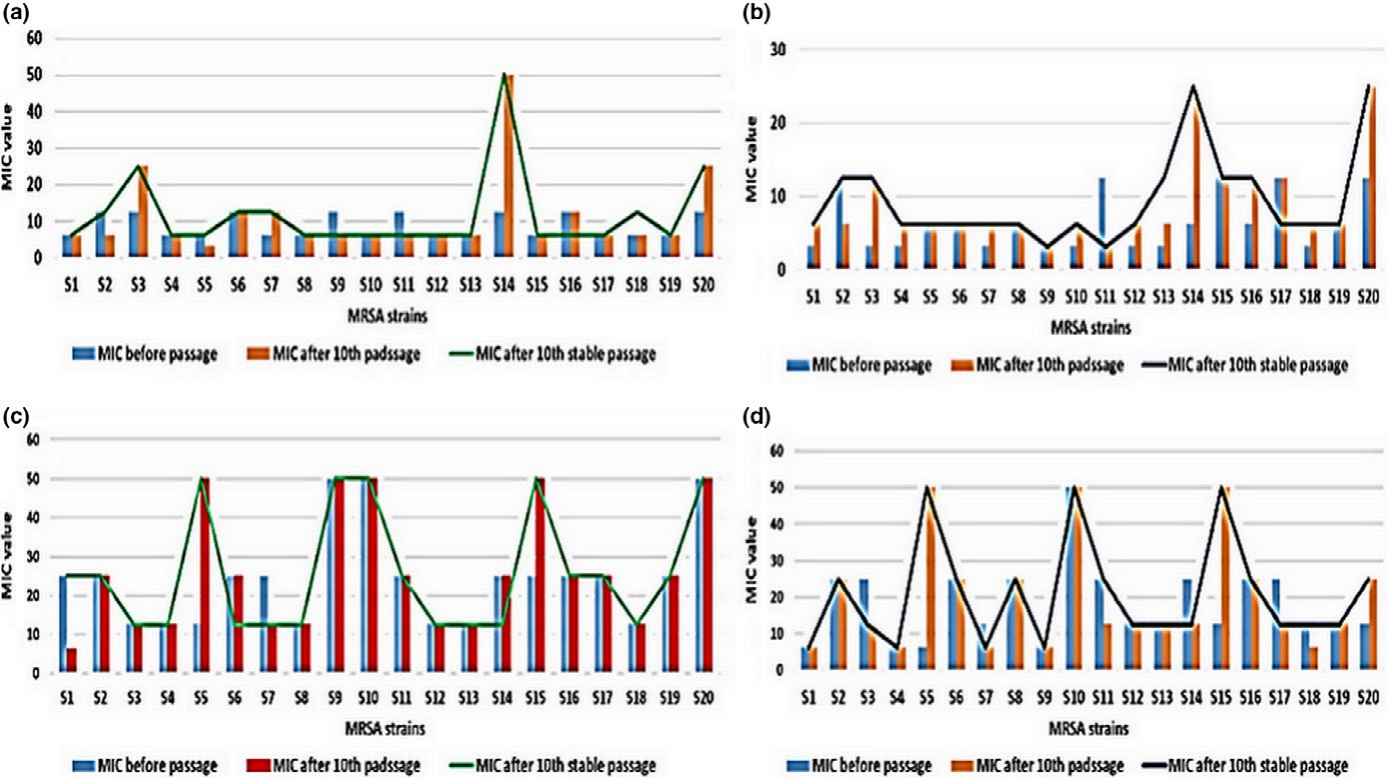
MIC of NPs against 20 strains of *S. aureus* before passage, after 10th passage, and after 10th stable passage. (a) 10‐nm AgNPs; (b) 20‐nm AgNPs; (c) 10‐nm AuNPs; and (d) 20‐nm AuNPs

The stability of the acquired resistance was observed in all AgNP‐ and AuNP‐adapted strains of *S. aureus* from the MIC after the 10th stable passage in NP‐free media (Figure 2). Overall, we observed, after the long‐term exposure of *S. aureus* to both NPs, that AuNPs (10 nm and 20 nm) induced less resistance in the majority of *S. aureus* strains than AgNPs, particularly 20‐nm AgNPs.

### 3.4 Probability of cross‐resistance between NPs and antibiotics

We next evaluated the sensitivity of 12 *S. aureus* strains (10 strains after long exposure to AgNPs and two strains after long exposure to AuNPs) to various antimicrobial agents commonly used for the treatment of *S. aureus* mastitis. As shown in Table 4, all strains showed high vulnerability to all of the antibiotics used in the study, except one of the *S. aureus* strains previously resistant to AgNPs and AuNPs, which showed strong resistance to benzyl penicillin and trimethoprim. The mean MIC values for benzyl penicillin and trimethoprim after the 10th stable exposure were 0.28 and 5.03 mg/L, respectively; otherwise, no resistance to the majority of the antibiotics used in this study was detected.

**Table 4:** The susceptibility NPs‐resistant *S. aureus* against antibiotics commonly used for treatment of bovine mastitis

Antibiotic	Epidemiological cut of value ECV≤	Clinical breakpoint (CB)>	Mean MIC (mg/L)			Resistance % for *S. aureus*		
			Before passage	After 10th exposure	After 10th stable exposure	Before exposure	After 10th exposure	After 10th stable exposure
Clindamycin	0.25	0.5	0.11	0.11	0.11	0	0	0
Erythromycin	1	2	0.5	0.53	0.5	0	0	0
Tetracycline	1	2	0.5	0.5	0.56	0	0	0
Ciprofloxacin	1	1	0.45	0.43	0.43	0	0	0
Rifampicin	0.016	—	0.016	0.016	0.016	0	0	0
Cifoxtin	4	4	1.1	1.06	1.06	0	0	0
Streptomycin	16	—	5.86	6.4	6.13	0	0	0
Thiamulin	2	0	1.13	1.13	1.13	0	0	0
Linezolid	4	4	1.66	1.8	1.8	0	0	0
Fusidic acid	0.5	1	0.5	0.5	0.5	0	0	0
Synercid	1	2	1.1	0.5	0.5	0	0	0
Mupirocin	0.5	—	0.5	0.5	0.5	0	0	0
Benzyl penicillin	0.12	—	0.37	0.36	0.28	0	8.33%	8.33%
Vancomycin	2	2	1.46	1.6	1.6	0	0	0
Sulphamethoxozole	128	—	88.7	85.76	85.74	0	0	0
Kanamycin	8	0	5.76	5.67	5.43	0	0	0
Trimethoprim	4	4	5.37	5.06	5.03	0	8.33%	8.33%

### 3.5 Adverse effects of AgNPs and AuNPs in rats in vivo

#### 3.5.1 Morphological changes in the rats

Some notable morphological changes in hair color (tan) were observed in the rats exposed to AgNPs (both 10 and 20 nm, 2 mg/kg dose). Furthermore, certain morphological changes were detected in the brain, liver, kidney, and heart of the G1 and G2 rats compared to controls (G5). Some morphological changes in the color and atrophy of the liver were observed in the treated G2 groups, which received 20‐nm AgNPs. To investigate the degree of the changes caused by the AgNPs and AuNPs, the following equation for the spleen index (Sx) was applied:

$$\text{Sx}=\frac{\text{Body weight of experimental organ/Body weight of experimental animal}}{\text{Body weight of control organ/Body weight of control animal}}$$

The mean value of Sx in the rat groups was 0.87 ± 0.19, close to 0.98 ± 0.20 (the control parameter obtained from G5). The appearance of the liver, kidney, and brain in the control group was nearly the same; however, G1 and G2 rats showed some dramatic changes in the color of the spleen (bronze color). These findings indicate that the spleen is one of the most prevalent target organs for NPs. No any other organs showed any gross morphological changes.

#### 3.5.2 Histopathological changes in the rats

The adverse effects of NPs were investigated after the oral administration of NPs to rats for 30 days. For the study doses of 0.25 mg, 0.5 mg, or 1 mg of AgNPs and AuNPs, the histopathological findings revealed no toxic effects in the various tested organs. In contrast, 2 mg/kg of either AgNPs or AuNPs was toxic to all examined organs.

**Figure 3:**
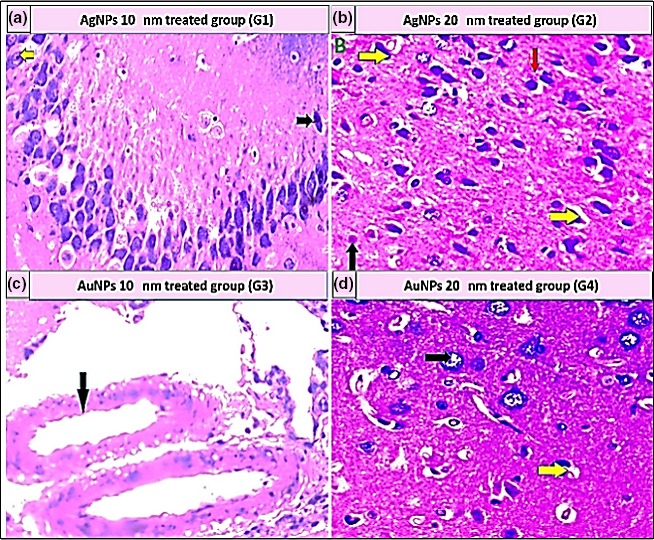
Histopathological examination of brain tissue stained with H&E (400×). (a) Aggregation of 10‐nm AgNPs in the neuropil (yellow arrow); some neuronal cells appeared darkly stained (dark arrow). (b) Distorted, darkly stained neurons (yellow arrows), mild apoptosis (black arrow), and 20‐nm AgNP deposition (red arrow). (c) Large blood vessels with hyalinization of their walls in the cerebral cortex of brain and deposition of 10‐nm AuNPs (black arrow). (d) Degenerative changes in the astrocytes (black arrow) with mild apoptosis (yellow arrows)

**Figure 4:**
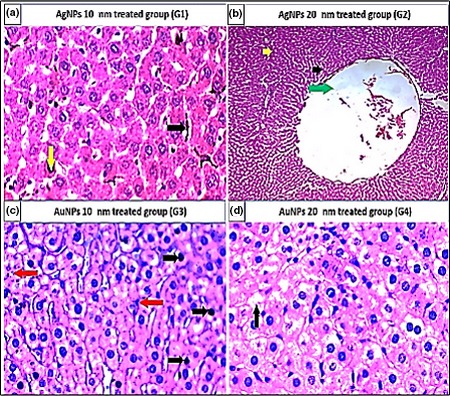
Histopathological examination of liver tissue stained with H&E (400×). (a) Apoptosis (black arrows) and NP pigment (yellow arrow). (b) Necrosis (green arrow), apoptosis (black arrows), and NP pigment (yellow arrow). (c) Apoptosis (black arrows) and NP pigment (red arrow). (d) Hydropic degeneration (black arrows)

**Figure 5:**
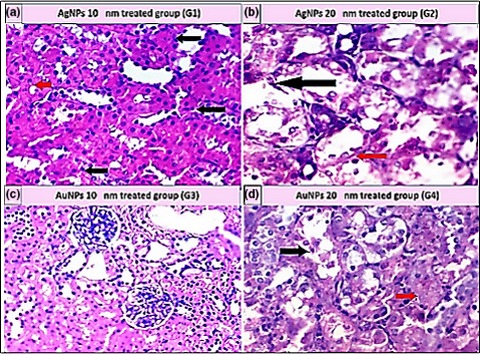
Histopathological examination of kidney tissue stained with H&E (400×). (a) Cloudy swelling (black arrow) and NP pigment (red arrow). (b) Marked tubular necrosis (black arrow) and NP pigment (red arrow). (c) Cloudy swelling (d) moderate tubular necrosis (black arrow) and NP pigment (red arrow)

**Figure 6:**
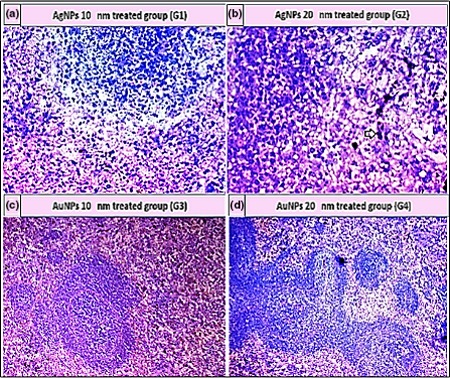
Histopathological examination of spleen tissue stained with H&E (400×). (a) NPs deposition. (b) Marked pigment deposition (black arrow). (c) Atrophied follicles, hemorrhage, and pigmentation. (d) Marked pigment deposition (black arrow)

**Figure 7:**
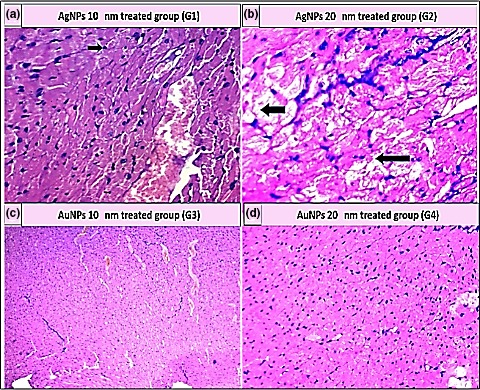
Histopathological examination of heart tissue stained with H&E (400×). (a) Apoptosis (black arrow) and blood vessel congestion (yellow arrow). (b) Marked hydropic degeneration (black arrow) and apoptosis. (c) Cloudy swelling and blood vessel congestion. (d) Hydropic degeneration and apoptosis

**Figure 8:**
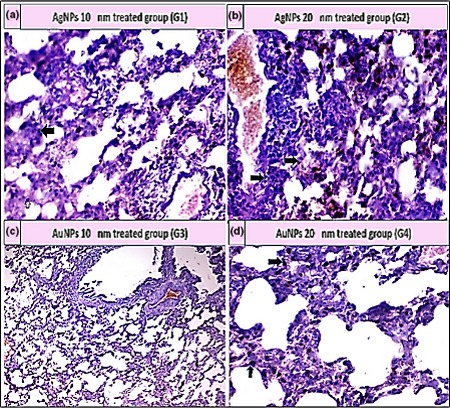
Histopathological examination of lung tissue stained with H&E (400×). (a) Moderate thickened interstitial septa and pigment deposition (black arrow); (b) Thickening of interstitial septa with marked pigment deposition (black arrow); (c) Mild thickened interstitial septa with mild pigmentation; and (d) Moderate thickened interstitial septa and pigment deposition (black arrow)

**Figure 9:**
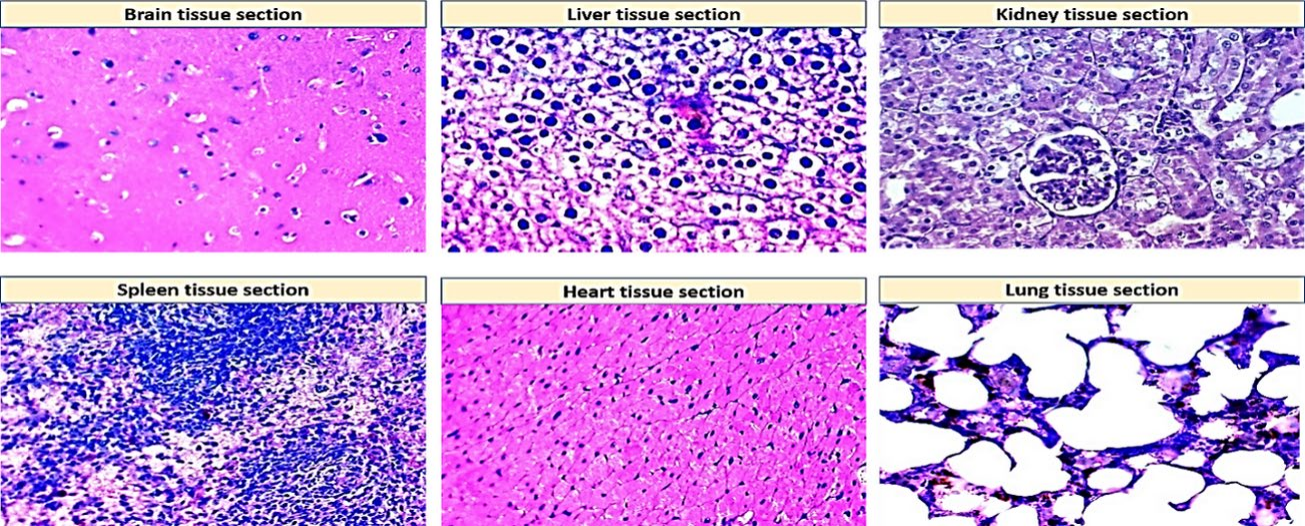
Histopathological examination of normal tissue sections for different organs (control group, G5) stained with H&E (400×)

As shown in Table 5, the histopathological examination showed that programmed cell death (apoptosis) and NP accumulation occurred in nearly all examined organs (Figures 3, 4, 5, 6, 7, 8), especially in the brain, which showed marked NP deposition in the neuropil of the brain tissue of all treated groups. Furthermore, severe hydropic degeneration and necrosis were observed in the hepatocytes of the liver (Figure 4). Moderate cloudy swelling (hydropic change or vacuolar degeneration) was detected in the kidney and heart tissues of all treated groups (Figures 5 and 7). Marked tubular necrosis and cloudy swelling were also found in the kidney tissue of the 20‐nm AgNP‐treated group (Figure 5b). In the lung tissue, moderate thickening of the interstitial septa was observed in G1, G3, and G4, while marked thickening was detected in G2 (Figure 8). As expected, the histopathological examination of the different organs of G5 revealed no pathological changes (Figure 9).

**Table 5:** The adverse effect of AgNPs and AuNPs at a concentration of 2 mg NPs/kg of body weight in rats

Target organ	Adverse effect of NPs			
	Group 1 (10‐nm AgNPs)	Group 2 (20‐nm AgNPs)	Group 3 (10‐nm AuNPs)	Group 4 (20‐nm AuNPs)
Brain	Distortion of neurons with darkly stained nuclei, Apoptosis, and NPs deposition	Aggregation of AuNPs in the neuropile, some neuronal cells appeared darkly stained shrunken	Astrocytes degeneration and apoptosis	NPs deposition, large‐sized blood vessels with hyalinization of their walls
Liver	Apoptosis and NPs pigment	Necrosis, apoptosis, and NPs pigment	Apoptosis and NPs pigment	Hydropic degeneration
Kidney	Cloudy swelling and NPs pigment	Marked tubular necrosis and NPs pigment	Cloudy swelling	Moderate tubular necrosis and NPs pigment
Heart	Apoptosis and congestion of blood vessels	Marked hydropic degeneration and apoptosis	Cloudy swelling	Hydropic degeneration and apoptosis
Spleen	Moderate pigment deposition	Marked pigment deposition	Atrophied follicles, hemorrhage, and moderate pigment	Marked pigment deposition
Lung	Moderate thickened interstial septa and moderate pigment deposition	Marked thickened interstial septa and marked pigment deposition	Moderate thickened interstial septa and mild pigmentation	Moderate thickened interstial septa and moderate pigment deposition

## 4 DISCUSSION

Abuse or misapplication of antibiotics has led to the relatively recent increase in multidrug‐resistant bacteria. To overcome the restrictions of traditional synthetic antimicrobial agents, nanotechnology approaches provide an alternative to antibiotics for the potential killing of mastitis pathogens (Dehkordi et al., 2011). Results from the serial dilution method showed that AgNPs have a strong antibacterial effect against the tested *S. aureus* isolates.

Similar results were shown in a study of Fernandez et al. (2008), where 9.8‐nm AgNPs at a concentration of 12.5 μg/ml were required to inhibit the growth of *S. aureus*. Martínez‐Castañón, Niño‐Martínez, Martínez‐Gutierrez, Martínez‐Mendoza, and Ruiz (2008) also revealed that 7‐nm and 29‐nm AgNPs were able to inhibit the growth of *S. aureus* at concentrations of 6.25 and 7.5 μg/ml, respectively. Moreover, Panáček et al. (2006) studied the antimicrobial effect of 25‐nm AgNPs against *S. aureus* and *E. coli*, and they found that 6.25–7.5 μg/ml AgNPs were needed to kill these bacteria.

In the work of Dehkordi et al. (2011), the antibacterial properties of 10‐nm AgNPs were studied against seven *S. aureus* strains that were isolated from subclinical bovine mastitis. The authors found that the MIC_50_ and MIC_90_ values were 5 μg/ml and 10 μg/ml, respectively. In addition, Hameed and El‐Zamkan (2015) revealed that synthesized AgNPs have a strong antimicrobial effect against *S*.* aureus* isolated from cheese samples.

The antibacterial activity of 10‐nm and 20‐nm AuNPs against *S. aureus* strains was also established in this study. Similar findings were obtained by Shamaila et al. (2016), who studied the antibacterial activity of AuNPs (7–34 nm in size) against *E. coli*,* Bacillus subtilis*,* S. aureus*, and *Klebsiella pneumonia* and found that the MICs of AuNPs against the tested bacteria were 2.93 μg/ml, 7.56 μg/ml, 3.92 μg/ml, and 3.15 μg/ml, respectively. In addition, Li et al. (2014) reported that various sizes of AuNPs successfully inhibited the growth of various multidrug‐resistant bacteria, including *S. aureus* and MRSA.

The mechanism underlying the antimicrobial action of AgNPs and AuNPs has previously been studied. It was suggested by Grier (1983) that the binding between NPs and components of the bacterial cell may cause impairment of the cell membrane, leading to cell death. In general, the action of AgNPs and AuNPs is parallel to that of silver and gold ions (Feng et al., 2000; Seil & Webster, 2012); they react not only with thiol groups and inhibit respiratory enzymes (Pal, Tak, & Song, 2007) but also with the sulfur and phosphorus groups of DNA, which are considered the most likely favorable sites for AgNPs (Russell et al., 1998) and AuNPs (Fricker, 1996). It can be seen that smallest NPs have the highest antimicrobial effect, compared to the larger NPs; this is due to their larger surface area‐to‐mass ratio that enhances their interaction with the bacterial surface. Although the antimicrobial activity of NPs is size dependent, the 10–20 size of NPs varies from one NP system to another (Rawashdeh & Haik, 2009). Recently, Lee and Lee (2018) studied the mode of action of AuNPs on *E. coli* and revealed that AuNPs induced bacterial apoptotic‐like cell death by rigorously damaging DNA. In addition, high levels of intracellular reactive oxygen species and reduced glutathione were observed in AuNP‐treated cells.

After inducing resistance in the *S. aureus* strains, we noted that the AuNPs (10 nm and 20 nm) failed to induce resistance to multiple *S. aureus* strains after 10 generations. In contrast, AgNPs, particularly 20‐nm AgNPs, induced a higher degree of resistance for 50% of the tested *S. aureus* strains. These results show AuNPs as better alternative method of treatment for bovine mastitis than AgNPs. The potent antibacterial effect of AuNPs as well as the lower induction of resistance in *S. aureus* can be explained by their aggregation within the biofilms of bacteria and their binding with the bacterial surface, which lead to alterations in the cell wall and decrease the period of treatment (Zawrah & Abd El‐Moez, 2011). Moreover, free radical release by AuNPs leads to bacterial cell death (Dakrong, Takuro, & Michael, 2011). The small size and the very large surface area of AuNPs can also help them to form holes in the bacterial cell wall by direct contact (Hazani et al., 2013). This disruption allows the intracellular contents to escape and to bind with the DNA, thereby preventing transcription (Rai, Prabhune, & Perry, 2010). The multiple mechanisms of action of AuNPs may explain the less‐frequent resistance to AuNPs obtained by *S. aureus*.

In contrast to large antimicrobial drugs, which commonly destroy bacteria through a single mechanism of action, NPs have characteristic sizes less than 100 nm. Their uniquely small size results in multiple innovative properties, for example, superior interaction with bacterial cells as a result of a larger surface area‐to‐mass ratio, and versatile and controllable application (Huh & Kwon, 2011); these properties make it difficult for bacteria to evolve a resistance to NPs.

However, some studies have indicated that NPs can induce bacterial resistance in special cases (Qiu et al., 2012; Wang et al., 2017). As AgNPs are known to share a similar mechanism of antimicrobial action with the silver ion (development of efflux systems), bacteria may create similar mechanism of resistance to NPs (Rawashdeh & Haik, 2009). Moreover, the stress response of bacteria from long‐term exposure must be considered.

The cross‐resistance of AgNP‐ and AuNP‐resistant *S. aureus* to various antimicrobial drugs was investigated in this study. In line with published reports, we did not find a link between resistance to NPs and antibiotic resistance; however, El Behiry et al. (2012), Karatzas et al. (2007), and Randall et al. (2007) tested the cross‐resistance between biocides and antibiotics and noticed that bacteria impervious to biocides were also impervious to antibiotics. In our study, the absence of any cross‐resistance suggests that the mechanisms that lead to NP resistance differ from those responsible for antibiotic resistance.

The toxic effects of small NPs were previously studied by Edwards‐Jones (2009), Nel, Xia, Mädler, and Li (2006), and Sardari et al. (2012). In this study, we investigated the toxicological effects of AgNPs and AuNPs in different organs (brain, liver, kidney, spleen, heart, and lung) after treating rats with various concentrations of NPs for 30 days (administered orally). The histopathological findings showed that AgNPs and AuNPs are not toxic at concentrations of 0.25, 0.5, or 1 mg/kg in the brain, liver, kidneys, heart, spleen, or lungs. In contrast, the higher dose (2 mg/kg) of NPs severely impaired all of the tested organs. As shown in Table 5, the most common adverse effects of NPs were apoptosis, NP deposition, cell necrosis, and tissue damage.

Similar findings were obtained by Sardari et al. (2012), who evaluated the potential toxicity of higher size AgNPs (70 nm) at different doses in rat tissues (liver, spleen, and kidney). They also found that adverse effects of AgNPs (tissue damage, bleeding, cell necrosis, and apoptosis) occurred at the 2 mg/kg dose. Park et al. (2010) studied the toxicological effects of AgNPs on rats after oral administration of three different sizes (22 nm, 71 nm, and 323 nm) of NPs. Treatment with 323‐nm AgNPs did not result in significant changes in the liver, kidney, or spleen, but treatment with 22‐nm or 71‐nm AgNPs resulted in AgNP absorption in the gastrointestinal system.

In another work conducted by Kim, Kim, Park, Ryu, and Yu (2009), it was noted that the administrated 60‐nm AgNPs at concentrations of 30, 300, or 1,000 mg/kg did not induce any significant changes in the body weight of rats; however, significant NP deposition was observed in different tissue organs and this accumulation was dose dependent. In addition, Park et al. (2010) and Weldon et al. (2016) studied the effects of 42‐nm AgNPs in rats that were given doses of 0.25 mg/kg, 0.5 mg/kg, or 1 mg/kg and found that long‐term oral administration of NPs induces liver toxicity in rats. These effects were explained by the high amounts of oxygen radicals produced from damage to the cell membrane, and the resulting organ failure.

Renal clearance of nanosized particles is extremely based on molecule size (Deen, Lazzara, & Myers, 2001). Particles with a hydrodynamic diameter less than 6 nm are completely filtered, whereas those more than 8 nm are not usually capable of glomerular filtration (Longmire, Choyke, & Kobayashi, 2008). In this work, we found that marked tubular necrosis of the kidney occurred after the rats received 10‐nm and 20‐nm NPs at 2 mg/kg. This necrosis was caused mainly by accumulation of AgNPs and AuNPs, which finally led to glomerular atrophy. Furthermore, some studies have found that silver ions are commonly distributed all over the body and finally aggregate in organs, especially the liver, spleen, and kidney (Lam, 2006; Oberdo, 1990; Sheng, Yi, Cheng, & Za, 2008). The histopathological changes in the brain, liver, spleen, kidneys, heart, and lung of rats that received AgNPs or AuNPs are indicative of silver and gold ions’ propensity to bind with thiol groups in the liver, induce reduction reactions, transfer glutathione to the gallbladder bile, and decrease the glutathione concentration. Hendi (2010) proved that the reduction in glutathione is very important to remove peroxides; consequently, various types of NPs can be toxic in both human and animal tissues, likely through this mechanism (Miura & Shinohara, 2009; Sardari et al., 2012). Future studies on the toxicity of NPs at different doses, shapes, and sizes are needed to ensure the safety and applicability of this promising technology.

## 5 CONCLUSIONS

The results of this study suggest that all of the *S. aureus* strains isolated from clinical and subclinical cases of mastitis exhibit significant susceptibility to AgNPs and AuNPs. The resistance developed by *S. aureus* against AgNPs was higher than that against AuNPs. Therefore, AuNPs represent an important complement to the antibiotics currently used in the prevention and treatment of bovine mastitis. Importantly, cross‐resistance between NPs and various antibiotics commonly used for the treatment of mastitis was not found in the majority of the tested *S*.* aureus* strains. Moreover, histopathological examination of various organs from treated rats shows that AgNPs and AuNPs are not toxic at low doses. In contrast, severe impairments of all tested organs were demonstrated at the higher dose (2 mg/kg) of NPs. The safety of NPs is a worldwide concern, and further studies must be carried out to clearly identify the biological effects of NPs.

## CONFLICT OF INTEREST

The authors declare that they have no competing interests to disclose.

## DATA ACCESSIBILITY STATEMENT

All data generated or analyzed during this study are included in this published article.
